# Obesity as the Main Risk Factor for Metabolic Syndrome in Children

**DOI:** 10.3389/fendo.2019.00568

**Published:** 2019-08-16

**Authors:** Vardit Gepstein, Ram Weiss

**Affiliations:** Section of Pediatric Endocrinology, Department of Pediatrics, Ruth Children's Hospital, Rambam Medical Center, Haifa, Israel

**Keywords:** metabolic syndrome (MetS), obesity, insulin resistance, childhood & adolescence, weight loss

## Abstract

Obesity in childhood is the main determinant of whole body reduced insulin sensitivity. This association has been demonstrated in multiple adult and pediatric cohorts. The mechanistic link explaining this association is the pattern of lipid partitioning in the face of excess calories and energy surplus. A tight relation exists between typical lipid deposition patterns, specifically within the skeletal muscle and liver, as well as the intra-abdominal compartment and whole body insulin sensitivity. The impact of lipid deposition within insulin responsive tissues such as the liver and skeletal muscle relates to the ability of fatty acid derivates to inhibit elements of the insulin signal transduction pathway. Strengthening the relation of obesity and reduced insulin sensitivity are the observations that weight gain reduces insulin sensitivity while weight loss increases it. This manifests as the appearance of cardiovascular risk factor clustering with weight gain and its recovery in the face of weight loss. Both obesity *per se*, via the adipocytokine profile it induces, and low insulin sensitivity, are independent determinants of the adverse metabolic phenotype characteristic of the metabolic syndrome.

The metabolic syndrome (MS), also known as “Insulin Resistance Syndrome” or “Syndrome X” describes clustering of established cardiovascular risk factors in specific individuals (1). These factors include altered glucose metabolism, elevated triglycerides, reduced HDL-cholesterol, elevated blood pressure and adiposity (1, 2) and have been shown to directly promote the development of atherosclerotic cardiovascular disease (3). While the exact definition of the syndrome in the pediatric age group is still debated, it is well-established that adults who meet the criteria for the syndrome are at increased risk for the development of type 2 diabetes (T2DM) and cardiovascular diseases over time, compared to individuals who do not meet these criteria. It was Gerald Reaven who first proposed the name “insulin resistance syndrome” to this risk factor clustering as he noticed and later demonstrated that adults with the metabolic syndrome tend to have lower insulin sensitivity (in other words—insulin resistance) compared to those who do not (4). Moreover, insulin resistance seems to be the major driving force of the development of the cardiovascular risk factors characteristic of the syndrome. Other factors such as local inflammation within relevant tissues and surrounding blood vessels feeding them and systemic subclinical inflammation may play a substantial role in the development of MS via inducing vaso-regulatory effects of local lipid deposits around blood vessels, which may contribute both to insulin action and endothelial dysfunction (5). In the presence of obesity, adipose tissue produces inflammatory cytokines in excess, whereas secretion of adiponectin is reduced highlighting the interplay between obesity and inflammation (6). Cardiovascular risk factor clustering (CVRFC, termed by some as the metabolic syndrome), is not a discrete entity with a single underlying cause and is probably the result of multiple underlying factors, yet the syndrome identifies individuals at an elevated risk for accelerated atherosclerosis.

The insights into the relevance of CVRFC in childhood stem from the established implications of such clustering in adulthood. The metabolic risk factors that are used to define the syndrome are those who have been shown to have a direct effect on atherosclerosis. There is a strong relation between the simple clinical markers of atherogenic dyslipidemia, namely elevated triglycerides and reduced HDL-cholesterol and the concentration of small LDL-cholesterol particles that carry the greatest risk of atherogenesis (7). Similarly, hyperglycemia and elevated blood pressure, even within the prediabetic or pre-hypertension levels, are also pro-atherogenic (8, 9). Importantly, while each component has been shown to increase cardiovascular risk their constellation increases the risk further. Several large longitudinal cohort studies performed in adults have shown greater cardiovascular morbidity and mortality in those having cardiovascular risk clustering compared to those who do not. For example, data from NHANES (10) in adults older than 50 years showed an odds ratio of 2.07 of cardiovascular disease for those who had such clustering compared to those who did not. Similarly, in the Framingham study—cardiovascular risk factor clustering predicted nearly 25% of cases of new onset cardiovascular disease over time (11). The DECODE study demonstrated that in adults without diabetes, cardiovascular risk factor clustering increases the risk of death from cardiovascular disease by 2.26 and 2.78 for men and women, respectively (12). Taken together, these observations in adults show that clustering of cardiovascular risk factor clearly increases the risk of cardiovascular disease over time. It is well-established that longer exposure to obesity in childhood increases the risk for the presence of such clustering (13) thus it is plausible to assume that such clustering in obese children increases their risk for earlier development of cardiovascular morbidity. As indicated above, the postulated mechanistic driving force of CVRFC in childhood as well as adulthood is insulin resistance.

Insulin resistance describes a reduced effect of insulin on its target tissues. This reduced effect may be limited to some tissues while being preserved in others and can also be specific to part of the insulin signal transduction pathway but not to other parts of it within the same tissue (14). For example, insulin receptors are widely distributed in the body in multiple tissues such as the traditional target organs liver and muscle as well as in tissues such as the kidney and the ovaries. In skeletal muscle, insulin's main role is to promote trafficking of the glucose transporter GLUT-4 to the cell membrane in order for glucose to enter into the myocyte. Muscle insulin resistance thus manifests as lower GLUT-4 expression on the membrane in response to insulin leading to reduced glucose uptake (15). Insulin resistance within the signal transduction pathway may be present in muscle while being entirely normal in the ovary (16). Within the liver, resistance may be present in in segments of the pathway relevant to glucose metabolism (such as suppression of glycogenolysis and gluconeogenesis) but not in those related to lipid metabolism and proliferation (17). This will manifest as reduced suppression of hepatic glucose production along with an increase of *de-novo* lipogenesis and VLDL production. In adipose tissue, the effect of insulin is to suppress lipolysis and adipose insulin resistance manifests as accelerated lipolysis (18).

There are multiple causes for the development of insulin resistance. These include a genetic background such as that observed in lean and healthy young adult offspring of patients with T2DM (19). An additional factor relevant to obese adolescents that contributes to transient reductions of insulin sensitivity is the pubertal period. The hormonal changes of puberty induce a ~33% reduction of whole body insulin sensitivity that reverts to basal levels upon sexual maturation (20). The effect of puberty on insulin sensitivity is suggested to be induced by growth hormone which causes increased lipolysis thus enhancing delivery of free fatty acids to skeletal muscle and liver. Supporting this hypothesis are the observations that in patients with growth hormone deficiency—insulin sensitivity is increased (21) while treatment with exogenous growth hormone in such patients reduces insulin sensitivity (22). Skeletal muscle contraction promotes GLUT-4 trafficking to the myocyte membrane independent of insulin thus accelerating glucose uptake (23). Increased physical activity, both aerobic and anaerobic, thus increases insulin sensitivity by this mechanism and by increasing skeletal muscle mitochondrial content (24) while lack of physical activity results in a marked reduction of insulin sensitivity (25). An additional factor that reduces insulin sensitivity is acute inflammation as observed during acute infections and trauma as well as the use of medications such as glucocorticoids (26, 27).

Of note, the relationship of BMI and insulin sensitivity has ethnicity-specific nuances. These nuances manifest- as differences in insulin sensitivity per comparable body habitus and as different degrees of CVRFC per given insulin sensitivity. For example—it has been shown that Hispanic adults are less insulin sensitive than Caucasians with similar BMI (28). Similarly, non-Hispanic Caucasians and African Americans have greater insulin sensitivity compared to Asians. In the same study, triglyceride levels were inversely associated with insulin sensitivity in all participants, yet for any given degree of insulin sensitivity, African Americans had the lowest triglyceride concentrations (29). These observations emphasize that while the mechanistic relations of obesity and insulin sensitivity and the impact of insulin sensitivity on CVRFC are universal in nature, their strength and metabolic manifestations differ by ethnicity and make individual patient assessments particularly tricky.

Taken together, the above mentioned factors may all contribute to reductions of insulin sensitivity in childhood to some degree yet they are typically not the main determining factor of the development of significant insulin resistance. The main factor associated with insulin resistance in childhood and adolescence is obesity.

## The Pathophysiology of Insulin Resistance and its Relation to Lipid Partitioning

It has been shown in multiple large cohorts across various ethnic backgrounds and age groups that insulin sensitivity is negatively correlated with body mass index (BMI, a crude measure of the degree of obesity). Having said that, a high BMI and severe obesity in childhood in an individual child do not necessarily confer the presence of very low insulin sensitivity. It is well-established that lipid partitioning, i.e., the pattern of lipid deposition, is a stronger determinant of whole body insulin sensitivity than the degree of obesity *per se* (30). Lipid partitioning in this context refers to the intracellular accumulation of lipid within cells of insulin responsive tissues such as the liver and skeletal muscle. Such intracellular accumulation renders cells vulnerable to the molecular effects of fatty acid derivates that may interfere with the normal insulin signal transduction pathway. Fat can be stored in extracellular depots such as the subcutaneous area and can also be stored within cells of insulin responsive tissues such as skeletal muscle and liver. An additional fat storage site is the intra-abdominal (visceral) compartment. In the context of energy surplus, lipid deposition within insulin responsive tissues, such as liver and muscle, has been shown to negatively affect the glucose related portions of the insulin signal transduction pathway (17). In this scenario, once the favorable fat depot (subcutaneous fat) exceeds its storage capacity, ectopic accumulation of lipid within the liver and skeletal muscle triggers molecular pathways that impair insulin signaling (15). In addition, storage of fat within the visceral compartment is associated with and adverse metabolic phenotype characterized by increased inflammatory cytokines further reducing insulin sensitivity and sub-clinical inflammation along with an accelerated flux of free fatty acids into the liver resulting in intra-hepatic lipid deposition (17). The absolute threshold above which normal lipid accumulation becomes pathological and induces adverse effects is unknown. Some propose that is it the ratio of visceral to subcutaneous fat rather than the absolute amount of visceral fat that determines the metabolic impact. Studies in adults who lost weight following bariatric surgical procedures hint to the presence of such threshold by showing that its level before weight loss is the strongest determinant of recovery from diabetes, dyslipidemia and hypertension during weight loss (31).

The presence of fat within the abdominal compartment and insulin responsive tissues represents part of normal physiology as it serves as an essential source of energy and heat production. Although not clearly defined, there probably is a threshold above which lipid accumulation within insulin responsive tissues or the intra-abdominal compartments turns from advantageous to deleterious (32). This theoretical threshold may be individual and based on the specific tissue capacity to metabolize lipids and their derivates. Upon passing this threshold, as typically observed in states of energy surplus such as childhood obesity, several molecular mechanisms within muscle, liver and adipose tissue explain the development of insulin resistance. The molecular mechanism leading to altered insulin-stimulated glucose transport in skeletal muscle and liver can be attributed to increases in intra-myocellular derived lipid metabolites such as fatty acyl CoAs and diacylglycerol (DAG) which activate a specific serine/threonine kinase cascade causing Ser/Thr phosphorylation of insulin receptor substrate (IRS)-1 and leading to defective insulin signaling (33). This leads to reduced skeletal muscle glucose uptake and to a reduction of liver glycogen synthesis and reduced suppression of gluconeogenesis. In most cases and specifically in childhood, skeletal muscle insulin resistance precedes the development of liver insulin resistance and leads to increased flux of intestinal derived circulating glucose to the liver. In the face of an increased glucose flux, the liver responds by increasing the process of *de novo* lipogenesis which leads to increased intrahepatic fat as well as greater circulating fatty acids and triacylglycerol. In parallel and independently, macrophage infiltration into white adipose tissue (intra-abdominal but also subcutaneous) results in adipose insulin resistance and leads to a shift in the balance toward greater lipolysis and lower lipogenesis. The liver thus faces an increased free fatty acid flux and this similarly leads to greater triglyceride synthesis and to systemic hyperlipidemia due to increased fatty acid esterification. The accelerated adipose tissue lipolysis and free fatty acid flux into the liver also causes stimulation of hepatic gluconeogenesis via activation of pyruvate carboxylase leading to under-suppressed hepatic glucose production manifesting as fasting as well as post-prandial hyperglycemia. As indicated above, insulin resistance within the tissues may develop as a result of intracellular accumulation of lipids. It has been shown in obese children that greater degree of obesity is associated with greater deposition of lipids within muscle (34) and liver (35) and that increasing obesity is associated with lower insulin sensitivity of adipose tissue itself (18). Importantly, macrophage infiltration of subcutaneous and intra-abdominal fat depots induces local and systemic sub-clinical inflammation and is tightly related to an adverse lipid partitioning profile in obese adolescents (36). Thus, in obese children and adolescents, the interplay of skeletal muscle, liver and adipose tissue insulin resistance, tightly linked to the lipid partitioning profile and mediated by signaling of multiple factors such as free fatty acids, leads to the development of glucose and lipid related alterations that are components of the metabolic syndrome.

**Figure 1:**
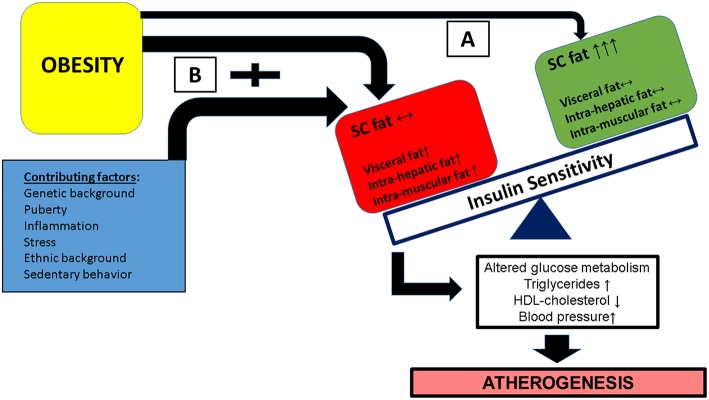
Obesity in childhood along with a contribution of additional factors (such as pubertal hormonal changes, specific ethnic backgrounds, exposure to stress among others) may have different metabolic/clinical results, based on the flux and deposition of excess lipid. **(A)** A favorable lipid partitioning pattern in which subcutaneous fat is capable of storing excess lipids and insulin responsive tissues (muscle and liver) are protected from excess lipid deposition. **(B)** Subcutaneous fat is incapable of storing excess lipid resulting in increased deposition of lipid in insulin responsive tissues leading to insulin resistance of these issues, specifically in pathways related to glucose metabolism. This results in the development of cardiovascular risk factor clustering (CVRFC) and manifests as accelerated atherogenesis.

As shown in Figure 1, the combination of multiple contributing factors (such as puberty, ethnic background, stress etc.) in the obese child along with an adverse lipid partitioning pattern characterized by incapacity of the subcutaneous fat to store excess lipid leading to intra hepatic, intra muscular, and visceral lipid deposition—leades to reduced insulin sensitivity and the development of cardiovascular risk factor clustering.

## Relation of Obesity in Childhood and Insulin Resistance

The relation of childhood obesity and insulin resistance to cardiovascular risk factor clustering (CVRFC), namely the metabolic syndrome, is well-established. Using a conservative definition of the syndrome, it was demonstrated that in a larger multi-ethnic cohort of obese children and adolescents, the prevalence of the syndrome independently increased with the degree of obesity as well as with rising degree of insulin resistance (37). Across a spectrum of BMIs, each half unit of the BMI z-score increased the risk of meeting the criteria of the syndrome by 55% (HR = 1.55, 95% CI 1.16–2.08). Independent of the degree of obesity, each additional unit of HOMA-IR (homeostatic model for assessment of insulin resistance, a surrogate of whole body insulin resistance calculated from fasting glucose and insulin concentrations) increased the risk for the presence of the syndrome by 12% (HR = 1.12, 95% CI = 1.07–1.18). Importantly, obesity and insulin resistance independent of each other within risk models were significant predictors of the presence of syndrome, highlighting their individual roles in its development. Using NHANES data of overweight and obese children and adolescents (38), it was shown that values for some, but not all, cardiovascular risk factors were higher in those with increasing severity of obesity. That study also showed that upon controlling age, race and sex, greater severity of obesity increased the risk for the presence of lower HDL-cholesterol levels, high systolic and diastolic blood pressure and elevated high triglycerides. In a population based study it has also been shown that adolescents within the upper 1 centile (>99% centile) of body mass index have a significantly greater risk for having cardiovascular risk factor clustering compared to those with lower degrees of obesity (39). In contrast, in an obesity clinic derived cohort (40) it was shown that indeed, greater adiposity is associated with greater risk of cardiovascular risk factor clustering yet that this risk tends to plateau in those with BMIs >40 kg/m^2^. This observation implies that up to a certain degree of adiposity, favorable lipid partitioning, namely greater subcutaneous rather than intra-abdominal fat depots, may allow maintaining relatively reasonable degrees of whole body insulin sensitivity. Above a certain threshold of adiposity, the limitations of lipid storage in the subcutaneous compartment are probably achieved and some degree of lipid deposition above the metabolically normal within insulin responsive tissues occurs. The ability of tissues such as liver and muscle to handle excess lipid deposition depends on the amount and performance of mitochondria within them. Importantly, even within an intracellular lipid deposition level that otherwise would be handled effectively without the development of significant deleterious effects on insulin signal transduction, “second hits” that affect these metabolic pathways may have a major impact. For example, pubertal maturation is a transient normal physiological process characterized by increased concentrations of growth and sex hormones. Puberty is known to transiently reduce whole body insulin sensitivity by ~30%, regardless of the degree of obesity (20). An obese child who already has some degree of insulin resistance yet is able to compensate appropriately by increasing insulin section and maintaining euglycemia may somewhat decompensate when entering puberty, where an additional metabolic burden occurs. Similarly, other factors that reduce insulin sensitivity such as exposure to corticosteroids or acute stress may unmask underlying insulin resistance that was previously well-compensated.

The impact of obesity on the development of insulin resistance and the presence of cardiovascular risk factors is not simply related to the presence but also to the length of exposure to excess adiposity. Specifically, earlier onset and longer duration of obesity are associated with greater risk for the presence of insulin resistance (13). Childhood obesity tends to track into adulthood, with up to 80% of obese children will eventually become obese adults (41), thus early onset obesity has a stronger impact on the deleterious potential effects of insulin resistance.

While the definitions of the metabolic syndrome in the pediatric age group are still in active debate, most analyses used them in large cohorts of children and adolescents with a full spectrum of anthropometric indices (from lean to morbidly obese). In the NHANES cohort—regardless of the definition used, the prevalence of the metabolic syndrome in lean adolescents was negligible (42). Similarly, the European IDEFICs group tested multiple definitions of the syndrome in a large cohort of children and showed that regardless of the definition used, the prevalence of the metabolic syndrome in lean children and adolescents is negligible (43). A recent Korean publication showed the same phenomenon in Korean adolescents, in which regardless of definitions used—lean children and adolescents did not meet criteria for the syndrome (44). These observations indicate that insulin resistance driven metabolic alterations are very rare in non-obese children and adolescents and further highlight the major role of obesity as the driver of the development of metabolic syndrome in childhood.

## Impact of Weight Dynamics on Insulin Sensitivity and Components of the Metabolic Syndrome in Obese Children

One would expect that if obesity is indeed the main cause of the presence of insulin resistance driven metabolic syndrome in children—weight loss should have a protective effect against the syndrome. Indeed, multiple studies have shown that weight loss achieved via lifestyle modifications (diet and exercise) leads to reversal of each component of the metabolic syndrome in childhood. Weight loss following lifestyle modification interventions has been shown to improve insulin sensitivity and to normalize several components of the syndrome. Multiple studies from across the world using a broad range of interventions that combine dietary modifications, physical activity and family therapy have demonstrated that that modest weight loss can result in a significant improvement of the metabolic phenotype. For example—a modest weight loss of ~7 kg in adolescents with a BMI of ~35 kg/m^2^ and HOMA-IR of ~5 at baseline resulted in a 1.5 unit HOMA-IR reduction (~30% improvement of insulin sensitivity) (45). Using OGTT derived indices of whole body insulin sensitivity demonstrated a similar magnitude of improvement (46). In a clinic based population of obese adolescents, it has been shown that a reduction of 0.30 BMI SD is associated with significant reductions of intra-hepatic and intra-muscular fat deposition (47) and tight and significant relation was demonstrated between BMI-z score reduction and insulin sensitivity increase (48). It has also been shown that an intensive intervention focused on exercise induces a weight reduction in obese children and adolescents and is associated improvement of insulin sensitivity but also an improvement of a clinical biomarker of atherogenesis such as intima-media thickness (49).

The above described studies suggest that the magnitude of BMI SD score reduction in obese adolescents is directly associated with the improvement in insulin sensitivity. The amount of weight needed to induce changes of insulin sensitivity that translate to improvement of clinical risk markers is usually in the range of 0.25 BMI SDs with those achieving >0.50 BMI SDs reduction showing the greatest benefit (50, 51). Importantly, multiple studies have shown that such improvements may be sustained several months following the completion of the program. The components of the metabolic syndrome that tend to improve most (together with insulin sensitivity) are fasting triglycerides, indices of glycemia and systolic blood pressure.

The significant long-term morbidity associated with obesity in the pediatric and adolescent population has also led to more aggressive treatment protocols, including surgical management. In parallel with the growing popularity of bariatric procedures in adult obese patients, there has been a growing body of evidence regarding their impact when performed in obese adolescents. These procedures differ in their relative combination of mechanical and hormonal effects yet can be evaluated in this context as a group in regards to their effects on body weight and whole body insulin sensitivity. In the Teen-LABS consortium prospective, multi-center, observational study, data from 242 adolescents reveled a significant weight loss (26–28%) post-surgery (gastric bypass and sleeve gastrectomy) that was associated with remission of diabetes in 95% of patients, and improvement in dyslipidemia (66%) and hypertension (74%) (52). Insulin resistance was not directly measured in this study yet using the surrogate of HOMA-IR showed a reduction of insulin resistance by a 3-fold magnitude (53). Similarly, a recent publication from Sweden (the AMOS study), reported the 5-year outcomes following RYGB surgery in adolescents, as compared with conservative treatment (54). This study also reported significant weight loss, which was maintained for 5 years and associated with a 74–100% resolution of the relevant comorbidities including in the incidence of diabetes, disturbed glucose intolerance, hypertension, dyslipidemia, inflammatory markers, and abnormal liver enzymes. Similar to TEEN-Labs, insulin sensitivity was not directly measured yet fasting insulin in this case dropped by a roughly 3-fold magnitude following the drastic weight loss. It should be noted that such clinical benefits should be balanced with the potential co-morbidities associated with surgical interventions such as the need for additional surgical interventions (25% of subjects in the AMOS study) and nutritional deficiencies (72%).

Weight gain is children provides the “other side of the coin” in regards to impact on whole body insulin sensitivity and its clinical correlates. In obese children, further weight gain is associated with a significant reduction in insulin sensitivity along with worsening of all components of the metabolic syndrome (55). Specifically, reductions in insulin sensitivity that were tightly and significantly related to weight gain emerged as the best predictors of deterioration of glucose tolerance (56). Importantly, longitudinal changes of insulin sensitivity are strongly related specifically to fat mass accrual over time (57), highlighting the mechanistic role of adipose tissue excess in the development of insulin resistance. The relation of weight dynamics and insulin sensitivity is well-described when intensive anti-obesity interventions are evaluated upon completion and their sustainability determined following a longer follow up. In such cases, weight loss immediately upon completion of a successful intervention parallels improvement of surrogates of insulin resistance (fasting insulin) yet upon weight regain on the later follow up—insulin resistance returns to its baseline level (58).

## Summary

Obesity in childhood is the main determinant of whole body reduced insulin sensitivity. Weight gain reduces insulin sensitivity and weight loss increases insulin sensitivity. Both obesity *per se* and low insulin sensitivity are independent determinants of the adverse metabolic phenotype characteristic of the metabolic syndrome. The impact of obesity on metabolism is modulated by the lipid partitioning patterns yet it is accurate to say that increased adiposity is associated in most children with some degree of insulin resistance and that it is the strongest predictor of the presence of cardiovascular risk factors in this age group.

## Author Contributions

All authors listed have made a substantial, direct and intellectual contribution to the work, and approved it for publication.

### Conflict of Interest Statement

The authors declare that the research was conducted in the absence of any commercial or financial relationships that could be construed as a potential conflict of interest.
